# The effectiveness of short mobile phone text message reminders compared to usual care on medication adherence in patients with hypertension: a systematic review protocol

**DOI:** 10.1186/s13643-023-02394-z

**Published:** 2024-02-05

**Authors:** Abebe Muche Belete, Daniel Molla Melese, Addisu Asefa, Yared Asmare Aynalem, Bekalu Bewket, Wondimeneh Shibabaw Shiferaw, Taklo Simeneh Yazie

**Affiliations:** 1 Department of Biomedical Science, Asrat Weldeyes Health Science Campus, Debre Berhan University, Debre Berhan, Ethiopia; 2 Faculty of Nursing, University of Alberta, Edmonton, Canada; 3 Department of Nursing, College of Health Science, Injibara University, Injibara, Ethiopia; 4 Faculty of Health, The University of Queensland, Brisbane, Australia; 5 Department of Pharmacy, College of Health Science, Debre Tabor University, Debre Tabor, Ethiopia

**Keywords:** Hypertension, Medication adherence, Text message, Usual care, Effectiveness

## Abstract

**Background:**

Poor adherence to long-term medication increases the risk of morbidity and mortality and decreases the quality of life of patients with hypertension. One strategy to improve treatment adherence is to use a short text message reminder. Although evidence indicates that such programs increase medication adherence, the extent of their effectiveness and translation into clinical practice needs to be better documented. Our systematic review will collect and analyze the available evidence for clinical practice implementation. This systematic review aimed to evaluate the effectiveness of short mobile phone text message reminders versus usual/standard care for medication adherence in patients with hypertension.

**Methods:**

This review will include and summarize evidence from randomized controlled trials. Adults (age > 18 years) with hypertension. The comparator group received either the usual care or standard care. It encompasses standard medical care for patients not participating in a structured and supervised intervention program such as a telemedicine program.

We will include studies that assess the effectiveness of short mobile phone text message reminders in improving medication adherence in patients with hypertension compared to usual care. We will search the following databases: PubMed, EMBASE, CINAHL, SCOPUS, Web of Science, Cochrane Library Central Register of Controlled Trials, and Cochrane Library. We will include studies published in English. Furthermore, we will consider studies published from the inception of the database until April 20, 2024. At least two reviewers will independently conduct study selection, data extraction, and quality assessment. A third reviewer will determine and resolve discrepancies. We will conduct a quality assessment using the ROBIS 2 critical appraisal checklist. At least two independent reviewers will crosscheck the data synthesis.

**Discussion:**

We expect this review to provide current evidence for future studies and clinical practice concerning the impact of mobile phone text message reminders on medication adherence issues. We will publish our results in a peer-reviewed journal for publication.

**Systematic review registration:**

PROSPERO CRD42023391236.

**Supplementary Information:**

The online version contains supplementary material available at 10.1186/s13643-023-02394-z.

## Background

Hypertension is a significant cause of cardiovascular disease and death worldwide, especially in low-income and middle-income countries [1]. An estimated 10% of global healthcare spending is directly related to increased blood pressure and its associated complications, including ischemic heart disease, heart failure, and stroke [2]. Control of hypertension reduces mortality and disability and is highly cost-effective or cost-saving in most settings [3]. Elevated systolic blood pressure is the leading cause of disability-adjusted life-years globally and in the Americas. Moreover, over 50% of ischemic heart disease events and strokes are attributable to high blood pressure [4]. The treatment of hypertension is also been shown to be highly cost-effective [5].

Poor adherence was the most common cause of resistant hypertension. Inadequate adherence to antihypertensive medication results in poorly controlled blood pressure and an increased risk of coronary and cerebrovascular events [6]. Missed appointments for the collection of medicines and challenges associated with lifelong treatment are among the significant reasons for suboptimal adherence [7]. Emerging technologies can be employed in interventions to improve adherence and reduce morbidity and mortality caused by poorly controlled hypertension [8]. Telemedicine allows clinicians to expand their reach by using technology to take care of patients who otherwise may not be seen [9–11]. Telemedicine presents an opportunity to increase medication adherence rates using electronic reminder systems [12]. They are an aid, particularly for patients suffering from forgetfulness problems and those who are unintentionally nonadherent. One example of telemedicine is a short mobile phone text message reminder system, which automatically sends reminder messages to a patient’s mobile phone in text messaging accessible on every mobile phone [13].

Adherence support delivered via short message system text messages can improve treatment adherence and health outcomes [14]. Text messaging is easy to use, widely accessible, and cost-effective. Moreover, it serves as a suitable intervention for various health behavior changes [15]. Hence, mobile phone‐based interventions are of particular interest because of their due to low cost and the potential for widespread delivery. However, no systematic review has specifically examined the effects of mobile phone text message interventions on adherence to antihypertensive medications. Therefore, the present review and meta-analysis aimed to evaluate the effect of mobile phone text message interventions on medication adherence among patients with hypertension and to provide applicable evidence for clinical practice.

A preliminary search of PROSPERO, MEDLINE, the Cochrane Database of Systematic Reviews, and JBI Evidence Synthesis was conducted. Unfortunately, no current or in-progress systematic reviews on the topic were identified.

### Review questions

What is the effectiveness of short mobile phone text message reminders versus usual care in improving medication adherence in people with hypertension?

## Methods

The proposed systematic review will be conducted per JBI methodology for systematic reviews of effectiveness [16] and is registered in PROSPERO [CRD42023391236].

### Inclusion criteria

#### Participants

This review will consider studies on medication adherence among hypertension patients aged ≥ 18 years who have received short mobile phone text message reminders and where there was an evaluation of medication adherence.

#### Interventions

This review will consider studies that evaluated short mobile phone text message reminders that measured medication adherence outcomes as part of the programs. For this review, the contents of reminders will be educational, behavioral, lifestyle, or other types of interventions.

#### Comparators

This review will consider studies comparing short mobile phone text message reminder interventions to usual or routine or standard care. Standard care/control is defined as usual care (including standard medical care or patients not participating in a structured and supervised intervention/program, such as a telemedicine program), self-care/self-education, or care received by unapproved/unqualified/uncertified/nonprofessional agents, such as family, caregivers, or friends.

#### Outcomes

This review will consider studies that evaluate medication adherence in patients with hypertension who attended text message reminders versus those who received usual care. This will be measured using the tools applied in those studies (e.g., self-report, Morisky’s Medication Adherence Scale (MMAS-8 or -4) or medication refill adherence or therapeutic drug monitoring).

#### Types of studies

This review will consider both experimental and quasi-experimental study designs, including randomized controlled trials; however, if randomized controlled trials are unavailable, quasi-experimental, case–control, and observational studies that include a comparator/control will be included to meet the objective of the study.

### Exclusion criteria

We will exclude studies that did not meet the inclusion criteria, those using text messages and phone call interventions and those lacking means and standard deviations for the meta-analysis.

#### Search strategy

The search strategy will aim to locate both published and unpublished studies. An initial limited search of PubMed and CINAHL was performed to identify articles on the topic. The text words contained in the titles and abstracts of relevant articles and the index terms used to describe the articles were used to develop a complete search strategy for PubMed (see Appendix). The search strategy, including all the identified keywords and index terms, will be adapted for each information source. The reference lists of all the studies selected for critical appraisal will be screened for additional studies. We will include studies published in English. We will also include studies published from the inception of the database until April 20, 2024.

We will search the following databases: PubMed, EMBASE, CINAHL, SCOPUS, Web of Science, Cochrane Library Central Register of Controlled Trials, and Cochrane Library. Additionally, we will search for sources of unpublished studies and gray literature, which include Google Scholar, ClinicalTrials.gov, ProQuest, thesis, and conference proceedings.

### Study selection

After the search, we will collect and upload all identified citations into Endnote software version 8 and then remove duplicates. After a pilot test, two independent reviewers will screen the titles and abstracts to assess them against the inclusion criteria for the review. We will retrieve potentially relevant studies in full and import their citation details into the JBI System for Unified Management, Assessment, and Review of Information [17]. Two independent reviewers will thoroughly assess the full text of the selected citations against the inclusion criteria. In addition, this systematic review will record and report reasons for excluding full-text studies that do not meet the inclusion criteria. If any disagreements arise between the reviewers at any stage of the study selection process, they will resolve them through discussion or by involving a third reviewer. We will fully report the results of the search and the process of selecting and including studies in the final systematic review, and we will present them using a Preferred Reporting Items for Systematic Reviews and Meta-Analyses-(PRISMA) flow diagram [18]. The checklist is available in Additional file 1.

### Assessment of methodological quality

Two independent reviewers will critically appraise eligible studies for methodological quality in the review. They will use standardized critical appraisal instruments from ROBIS 2 [19] for experimental, quasi-experimental, and observational studies. If required, we will reach out to the authors of papers to seek missing or additional data for clarification. We will resolve any disagreements between the reviewers through discussion or by including a third reviewer. We will present the results of the critical appraisal in a table along with a narrative description.

All studies, regardless of the results of their methodological quality results, will undergo data extraction and synthesis whenever possible. Finally, we will summarize the critical appraisal results and incorporate them into the review in table format.

### Data extraction

Data will be extracted from studies included in the review by two independent reviewers using the standardized JBI data extraction tool [20]. The data extracted will include specific details about the populations, study types and methods, mobile text messages, adherence measurement tools, and outcomes of significance to the review question. For example, the mobile text message includes the amount of messaging, the content of the message, received reminders, motivational and supported messages, and advice on lifestyle behaviors like diets, physical activity, medication, and appointment reminders. Regarding the intervention, we will extract frequency, start and duration, measures of adherence to the intervention, and adherence to antihypertensive medication. Any reviewer disagreements will be resolved through discussion or with a third reviewer. Authors of papers will be contacted to request missing or additional data, where required.

### Data management

If data is missing, we will reach out to investigators to obtain the required information. Furthermore, our plan includes contacting investigators to obtain any missing data, especially in cases where a study is only available as an abstract. If it is not possible to contact investigators and missing data might introduce significant bias, we will conduct a sensitivity analysis to evaluate the impact of including such studies in the overall results assessment. In situations where measures of variation are still unavailable or the data display skewness, we will describe the available data narratively.

### Data synthesis

We will pool studies using statistical meta-analysis using the Review Manager of the Cochrane Collaboration (RevMan 5.4, Cochrane Organization). Effect sizes will be expressed as either odds ratios (for dichotomous data) or weighted (or standardized) final post-intervention mean differences (for continuous data), and their 95% confidence intervals will be calculated for analysis. We will evaluate heterogeneity using standard statistical tests including *χ*^2^ and *I*^2^ tests [21]. Statistical analyses will be performed using a random effect model [22]. Subgroup analyses will be conducted with sufficient data to investigate thematic areas such as adherence tools and settings. Finally, sensitivity analyses will be conducted to test the decisions made regarding the effectiveness of an intervention on the outcome of the study. Where statistical pooling is not possible, the findings will be presented in narrative form, including tables and figures, to aid in data presentation, where appropriate.

A funnel plot will be generated using RevMan 5.0 (Copenhagen: The Nordic Cochrane Centre, Cochrane to assess publication bias if there are ten or more studies included in a meta-analysis. In addition, statistical tests for funnel plot asymmetry (Egger test, Begg test, Harbord test) will be performed where appropriate.

### Assessing certainty in the findings

The Grading of Recommendations, Assessment, Development and Evaluation (GRADE) approach for grading the certainty of evidence will be followed [23], and a Summary of Findings will be created using GRADE pro-GDT (McMaster University, ON, Canada). The Summary of Findings will present the following information where appropriate: absolute risks for the treatment and control, estimates of relative risk, and a ranking of the quality of the evidence based on the risk of bias, directness, heterogeneity, precision, and risk of publication bias of the review results. The outcomes reported in the Summary of Findings will be medication adherence.

## Discussion

Medication adherence is a top priority in hypertension management. Therefore, it is necessary to analyze the effectiveness of short mobile phone text message reminders on medication adherence among adult patients with hypertension. Our results will be used to inform healthcare providers, policymakers, patients with hypertension, and family members of the relative effectiveness of short mobile phone text message reminders. We will implement rigorous and evidence-based knowledge and translation strategies to ensure that our results reach key stakeholders such as policymakers, doctors, and patients.

### Strength and limitation

This review has several strengths. To improve the quality of the review, we will employ a pre-defined methodology based on the Cochrane Handbook for Systematic Reviews of Interventions. Additionally, we will assess the strength of evidence based on GRADE. The review protocol’s strengths lie in its systematic and evidence-based approach. By conducting a systematic review, our research team ensures a comprehensive and thorough examination of the available literature. This approach allows for the synthesis of multiple studies, providing a more robust and generalizable conclusion about the effectiveness of mobile phone text message reminders on medication adherence in hypertension patients. It contributes to the generation of evidence-based recommendations that can inform clinical practice and healthcare policy.

However, this review has some limitations. This may have introduced bias by including only studies published in English. Additionally, the review did not include an analysis of the cost-effectiveness of short mobile phone text messages.

### Supplementary Information

**Additional file 1.** PRISMA check.

## Data Availability

All data generated or analyzed during this study are included in this published article.

## Appendix

**Table 1:** Search strategies using PubMed database from January 2000 to February 5, 2023

Search number	Query	Results
1	Hypertension, blood pressure	206,741
2	Female, male, adult, middle-aged, aged, older	153,260
3	1 and 2	4271
4	Short message OR message system OR SMS OR text messaging OR mobile phone text message OR text message reminder OR reminder systems OR digital health OR telemedicine	136,918
5	3 AND 4	35
6	Treatment adherence/or treatment compliance/or patient acceptance of health care or patient compliance/or medication adherence/	397,913
7	Medication or drug or pharmacological or therapeutic or treatment or adherence or compliance or concordance	19,445,227
8	Patient medication or drug or pharmacological or therapeutic or treatment or knowledge or understanding or awareness or adherence or compliance or attitude or practice	18,428,612
9	6 or 7 or 8	20,428,855
10	5 and 9	33
11	Double-blind method/or clinical trials/or clinical trials, phase I as topic/or clinical trials, phase ii as topic/or clinical trials, phase iii as topic/or clinical trials, phase iv as topic/or controlled clinical trials as topic/or randomized controlled trials as topic/or early termination of clinical trials topic/or multicenter studies as topic/	1,517,297
12	(randomized adj4 trial) or (controlled adj3 trial) or (clinical adj2 trial) or ((single or double or tripl or treb) and (blind or mask)) or ("4 arms" or "four arms")	62,328
13	"clinical trial" or "clinical trial, phase i" or "clinical trial, phase ii" or clinical trial, phase iii or clinical trial, phase iv or controlled clinical trial or "multicenter study" or "randomized controlled trial."	1,457,599
14	11 or 12 or 13	1,823,699
15	10 and 14	15

## Abbreviations

GRADE: Grading of Recommendations, Assessment, Development and Evaluation
JBI: Joanna Briggs Institute
PRISMA: Preferred Reporting Items for Systematic Reviews and Meta-analyses
ROBIS 2: Risk of Bias Assessment 2
